# Calcified mucinous adenocarcinoma of the stomach metastatic to the iris: a case report

**DOI:** 10.1186/s13256-019-1977-z

**Published:** 2019-03-07

**Authors:** Miki Kaneko, Tadashi Namisaki, Hiroaki Takaya, Hitoshi Mori, Mitsuteru Kitade, Yasushi Okura, Kenichiro Seki, Shinya Sato, Keisuke Nakanishi, Koh Kitagawa, Takahiro Ozutsumi, Naotaka Shimozato, Kosuke Kaji, Tomoyuki Otani, Tokiko Nakai, Chiho Obayashi, Akira Mitoro, Junichi Yamao, Hitoshi Yoshiji

**Affiliations:** 1 Third Department of Internal Medicine, Nara Medical University, 840 Shijo-cho, Kashihara, Nara 634-8522 Japan; 2 Department of Diagnostic Pathology, Nara Medical University School of Medicine, Nara, Japan

**Keywords:** Gastric cancer, Mucinous adenocarcinoma, Iris metastasis, Calcification, Chemotherapy

## Abstract

**Background:**

Gastric cancer has a wide spectrum of clinical features, imaging manifestations, and pathology. Punctate calcifications in gastric cancer are infrequent but are usually found in mucinous adenocarcinoma. However, there have only been a few autopsy case reports describing the correlation between the radiology and pathology findings of calcified mucinous adenocarcinoma of the stomach. We present an autopsy case of mucinous gastric adenocarcinoma with iris metastases as the initial symptom.

**Case presentation:**

A 74-year-old Japanese woman presented with blurred vision. Her treating ophthalmologist diagnosed acute iritis with secondary glaucoma. The histopathological and immunohistochemical features of a trabeculectomy specimen favored metastatic carcinoma, most likely of gastrointestinal tract origin. Esophagogastroduodenoscopy revealed multiple irregularly shaped ulcerative lesions, multiple erosions, and thickened folds in the corpus of her stomach. Histologic examination of a gastric tissue specimen obtained by endoscopic biopsy revealed poorly differentiated carcinoma with signet ring cell features. Computed tomography revealed a tumor with multiple punctate calcifications in the thickened gastric wall with diffuse low attenuation and multiple lymph node metastases, including the para-aortic lymph nodes, and peritoneal dissemination. She was diagnosed with stage IV gastric cancer (T4N3M1) and underwent seven cycles of 5-weekly TS-1, a novel oral fluoropyrimidine derivative, plus cisplatin therapy. Serial follow-up computed tomography revealed successive increases in the gastric wall calcifications. Her disease stabilized, but she died of aspiration pneumonia 8 months after the first visit. Autopsy tissue specimens had miliary, punctate calcifications present in abundant extracellular mucin pools in the submucosa, corresponding to the thickened low-attenuating middle layer on computed tomography. The final diagnosis was mucinous gastric adenocarcinoma because mucinous adenocarcinoma is diagnosed when more than half of the tumor area contains extracellular mucin pools.

**Conclusions:**

We report the pathology and computed tomography imaging characteristics of a case of calcified mucinous adenocarcinoma of the stomach metastatic to the iris, including findings at autopsy. Metastatic carcinomas in the iris originating in the stomach are exceedingly rare. Multiple punctate calcifications were present in pools of extracellular mucin, a diagnostic clue for mucinous adenocarcinoma. Possible mechanisms underlying scattered punctuate calcifications in gastric mucinous adenocarcinoma warrant further investigation.

## Background

Gastric adenocarcinoma is the fifth most common cancer and third leading cause of cancer-related death in the world [1]. The incidence of and mortality from gastric cancer have fallen dramatically over the past several decades. Gastric adenocarcinoma commonly develops slowly, but if not recognized, it may eventually result in overt metastases in multiple organs [2]. Ocular metastasis from carcinomas has been reported to occur in 4 to 8% of cases in autopsy series [3, 4]. The choroid is the most frequent site of ocular metastasis [5], whereas metastatic spread to the iris is exceedingly uncommon [2, 6–8]. Metastatic tumors to the iris generally originate from primary malignancies in the breast, lung, skin, kidney, and esophagus [6].

Gastric cancer has a wide spectrum of clinical features, imaging manifestations, and pathology. Calcifications in gastric cancer are infrequent but are usually found in mucinous adenocarcinoma [9], where diffuse, punctate calcifications are observed [10, 11]. Most previously reported cases of calcified gastric cancer were histologically confirmed as a mucinous adenocarcinoma on endoscopic biopsy or surgical specimens. However, there have only been a few autopsy case reports describing the correlation between the radiology and pathology findings of calcified mucinous adenocarcinoma of the stomach. We report the pathology and computed tomography (CT) imaging characteristics of a case of calcified mucinous adenocarcinoma of the stomach metastatic to the iris, including findings at autopsy.

## Case presentation

**Fig. 1:**
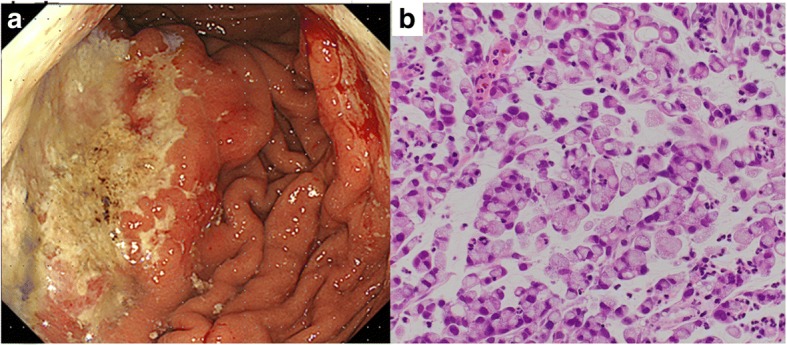
Esophagogastroduodenoscopy and pathology findings in gastric mucinous adenocarcinoma. **a** Esophagogastroduodenoscopy view of an advanced gastric cancer in the anterior wall of the gastric corpus. **b** The gastric biopsy specimen reveals poorly differentiated adenocarcinoma with signet ring cells (hematoxylin and eosin stain, × 40)

**Fig. 2:**
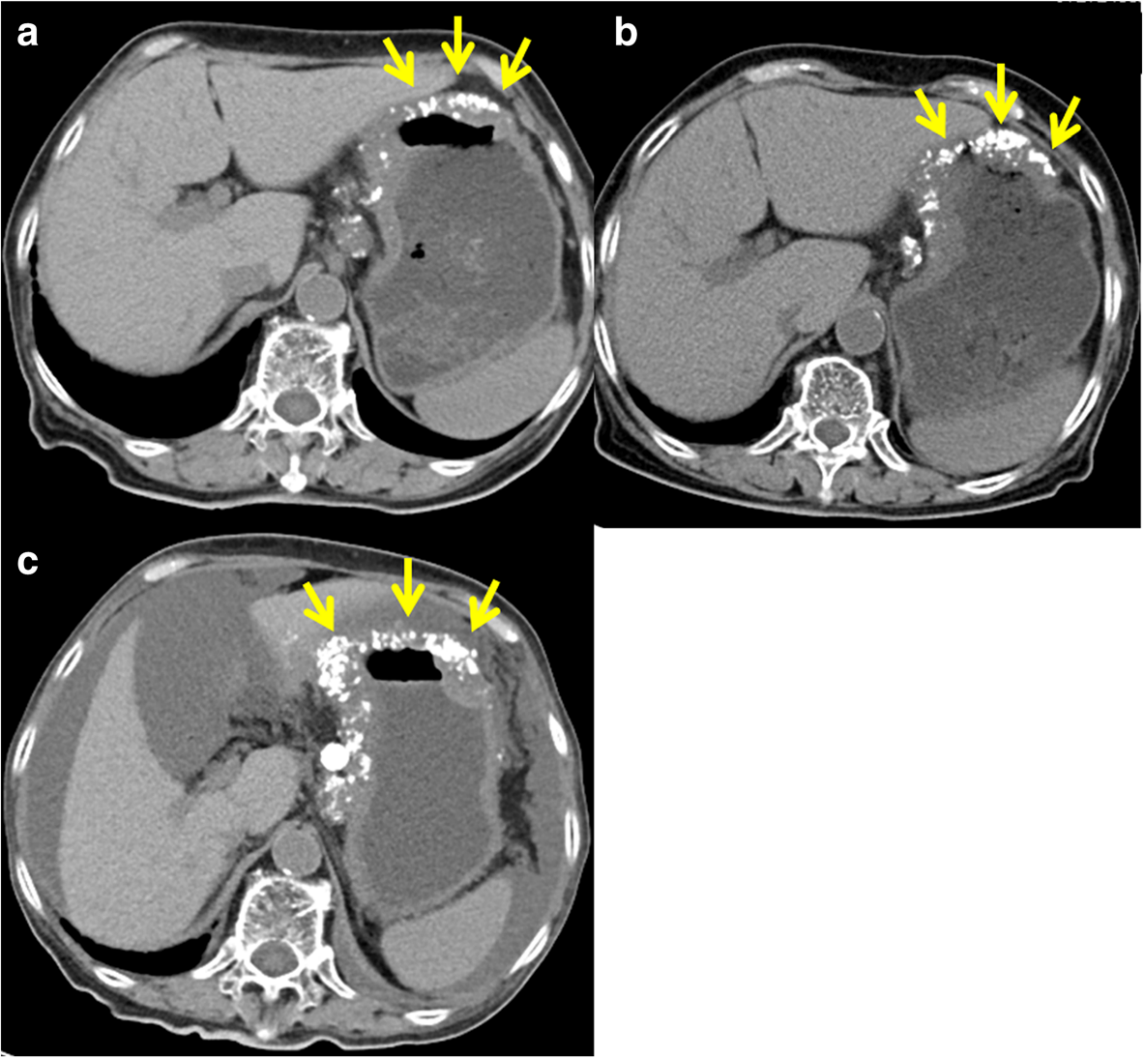
Contrast-enhanced computed tomography images of mucinous gastric carcinoma. There is diffuse low attenuation in the thickened gastric wall along with punctuate calcifications (*yellow arrows*), which increase in number with successive images. **a** Prior to chemotherapy. **b** After three cycles of chemotherapy. **c** One month after chemotherapy ends. Massive ascites has accumulated within the peritoneal cavity

A 74-year-old Japanese woman presented with a chief complaint of blurred vision and elevated intraocular pressure in her right eye during the previous month. She had a 20-year history of type 2 diabetes and hypertension. She had no known cancer, malignant lymphoma, or ocular manifestations of cancer. Her physical examination findings were unremarkable. Her treating ophthalmologist diagnosed acute iritis with secondary glaucoma. She underwent a trabeculectomy because topical corticosteroids and antiglaucoma medications had been ineffective in lowering the intraocular pressure. However, postoperatively, she still had diffuse thickening of the iris and white masses resembling frog spawn in the anterior chamber. An iris biopsy was performed, and immunocytochemistry analysis showed that the tumor cells were positive for cytokeratin (CK)-CAM5.2 and CDX2 and negative for CK7, CK20, thyroid transcription factor 1 (TTF-1), and anaplastic lymphoma kinase (ALK). These findings indicated a primary epithelial tumor, most likely from the gastrointestinal tract. She underwent positron emission tomography/CT to locate a primary tumor, but no abnormality was seen. Esophagogastroduodenoscopy demonstrated multiple irregularly shaped ulcerative lesions, multiple erosions, and thickened folds in the corpus of her stomach (Fig. 1a). A biopsy of a gastric tissue specimen revealed poorly differentiated carcinoma with signet ring cell features (Fig. 1b). CT revealed diffuse, low attenuation thickening of the gastric wall with punctuate calcifications (Fig. 2a). There were metastases to the para-aortic and mesenteric lymph nodes and peritoneal seeding. She was diagnosed as having poorly differentiated gastric adenocarcinoma metastatic to the iris, peritoneum, and lymph nodes.

**Fig. 3:**
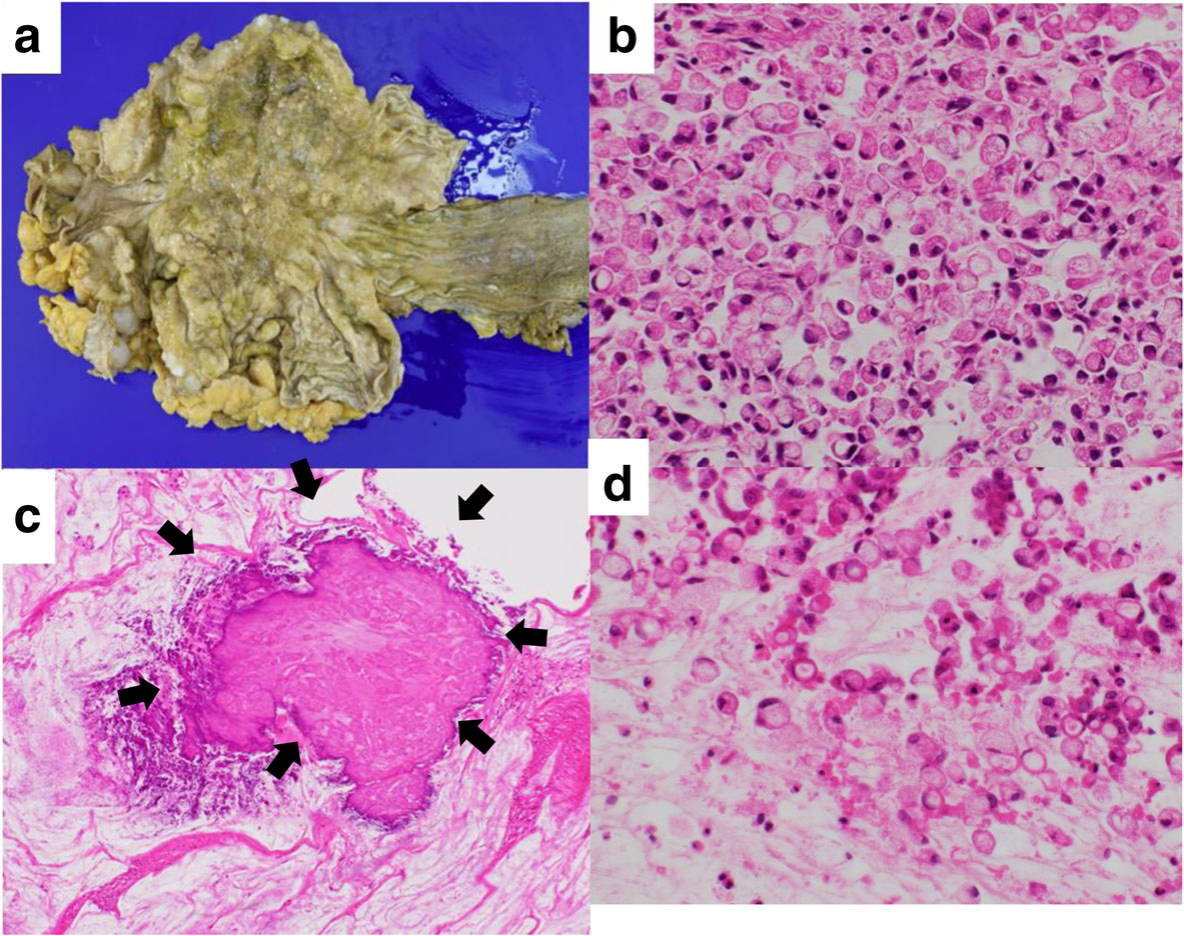
Gross pathologic and microscopic findings of the stomach at autopsy. **a** An ulcerated tumor has invaded through the wall of the stomach and into the esophagus. **b** Histology demonstrates a signet ring cell adenocarcinoma with (**c**) calcifications (*black arrows*) in a mucin pool (hematoxylin and eosin stain, × 10). **d** Histology shows scattered signet ring cells floating in the abundant mucin (hematoxylin and eosin stain, × 10)

She received a total of seven courses of TS-1, a novel oral fluoropyrimidine derivative that comprises the 5-fluorouracil prodrug tegafur (Ftorafur, FT) and two enzyme inhibitors, 5-chloro-2,4-dihydroxypyridine (CDHP) and potassium oxonate (OXO) in a molar ratio of 1(FT):0.4 (CDHP):1(OXO) (40 mg/m^2^, twice a day, on days 1–21) and intravenously administered cisplatin (60 mg/m^2^, on day 8) every 5 weeks (SPIRITS regimen) [12]. The white masses in the anterior chamber had slightly diminished after three cycles of chemotherapy and remained stable for seven cycles. A CT scan after six cycles of chemotherapy revealed no evidence of disease progression, although serum carcinoembryonic antigen levels gradually increased from 6.8 ng/mL at diagnosis to 28.4 ng/mL after six cycles. After seven cycles, CT revealed massive ascites that had accumulated since cycle 6. Serial follow-up CT revealed successive increases of calcifications in the gastric wall during the course of chemotherapy (Fig. 2b, c). She ultimately died of aspiration pneumonia 8 months after presentation. An autopsy revealed an ulcerated, invasive tumor involving the entire thickness of the wall of the stomach (Fig. 3a). It had also spread into the esophagus and the para-aortic and mesenteric lymph nodes. Mucinous adenocarcinoma is diagnosed when more than half of the tumor area contains extracellular mucin pools; signet ring cell carcinoma is diagnosed when adenocarcinoma is seen with a predominant component (> 50%) of isolated tumor cells that contain mucin [13]. Histologic examination revealed poorly differentiated adenocarcinoma containing signet ring cells beneath a preserved surface epithelium (Fig. 3b) and calcifications among the mucous lakes in the deep layers (Fig. 3c). Other areas showed scattered signet ring cells floating in the abundant mucin (Fig. 3d). The final diagnosis was mucinous gastric adenocarcinoma metastatic to the iris, peritoneum, and lymph nodes. The calcifications were present in extracellular mucin pools in the submucosa, corresponding to the thickened, low-attenuating middle layer seen on CT.

## Discussion

Calcifications in gastric cancer are an uncommon finding, but when present, they are usually associated with mucinous adenocarcinoma. In the current case, multiple punctate calcifications were present in pools of extracellular mucin, a diagnostic clue for mucinous adenocarcinoma. Metastatic carcinomas in the iris that originate from the stomach are exceedingly rare. To the best of our knowledge, this is the first autopsy case report of gastric mucinous adenocarcinoma metastatic to the iris.

Several different types of calcification are associated with varying pathologic characteristics, including those seen in mucin pools as in our patient, psammomatous calcifications, and heterotopic ossification [14]. Among these, mucin pool calcifications are relatively frequently seen [15]. The miliary, punctate calcifications seen in mucin-producing gastric adenocarcinoma can be distinguished from the circumscribed and patchy calcification found in benign tumors such as leiomyomata and hemangiomas [16]. Psammomatous calcifications are associated with non-mucin-producing carcinomas [17], particularly in gynecologic serous malignancies [18–20]. Such psammomatous calcifications have been reportedly scattered within the tumor stroma and glandular lumina in colon cancer [21]. They have also been observed in metastases to the ovary from primary mucin-producing tumors of the gastrointestinal tract (that is, Krukenberg tumor). However, these calcifications are sometimes difficult to detect on X-rays or ultrasound due to their small size [17]. Heterotopic ossification may occur in well-differentiated primary and metastatic adenocarcinoma [22].

The type of calcification observed on CT imaging of malignancies has been described as dystrophic or metastatic, suggesting differences in the pathogenesis of the calcification [14]. Dystrophic calcification is observed with tissue necrosis caused by chemotherapy, whereas metastatic calcification occurs with disorders of mineral balance, such as with hyperparathyroidism or uremia [23]. In our patient, calcifications increased after chemotherapy was initiated. At autopsy, the calcifications were histologically confirmed to be located within the necrotic areas of a tumor nest. Wang *et al.* reported the case of a patient with signet ring gastric cancer in which the amount of calcification observed on CT increased even as the patient responded to chemotherapy with a decrease in the gastric wall thickness [15]. They speculated, as had been suggested by Rotondo *et al*. [16], that dystrophic calcification in ischemic and necrotic areas generated by chemotherapy occurs because of the relative alkalinity of the surrounding extracellular fluid. A plausible explanation for these findings is that chemotherapy creates areas of ischemia and necrosis due to insufficient blood flow, reducing the carbon dioxide content in the blood and during cellular respiration, a process contributing to relative alkalinity [24]. This alkalinity of the blood or tissue fluids favors calcification [16, 25].

In contrast to the observed increases in calcification, Balestreri *et al*. reported the case of a patient with calcified signet ring cell gastric cancer in whom the calcification gradually decreased following chemotherapy [14]. Mucin pool calcifications observed in mucinous carcinoma, by definition, exhibit prominent mucin production that is observed in extracellular pools [26]. The glycoprotein mucin functions as an ion-exchange resin, such that the accumulation of mucin in the tumor and its metastases may augment the deposition of calcium [27, 28]. The decrease in calcifications on CT after chemotherapy would then be more likely to be associated with a decrease in the amount of mucin.

Gastric adenocarcinoma metastasizing to the iris is extremely uncommon. Among metastatic tumors in the iris, gastrointestinal tract carcinoma was the primary site in only 9% of cases [2, 5]. In 13 (32%) of 40 patients with metastases to the iris, blurred vision preceded the diagnosis of the primary tumors [29]. Any patient with a new onset of ophthalmic symptoms and a mass in the eye should be carefully evaluated for a primary tumor metastatic to the eye.

## Conclusions

We present the case of a patient with mucinous gastric adenocarcinoma with signet ring cell features who presented with iris metastases as the initial symptom. Findings at autopsy were correlated with CT images during diagnosis and treatment. Scattered punctate calcifications within the mucin pool are a clue to the diagnosis of mucinous carcinoma. Possible mechanisms underlying the calcifications in gastric mucinous adenocarcinoma may still warrant further investigation.

## Abbreviations

CDHP: 5-chloro-2,4-dihydroxypyridine
CK: Cytokeratin
CT: Computed tomography
FT: Tegafur (Ftorafur)
OXO: Potassium oxonate
